# A Clinical Investigation on Serum Amyloid A Concentration in Client-Owned Healthy and Diseased Cats in a Primary Care Animal Hospital

**DOI:** 10.3390/vetsci7020045

**Published:** 2020-04-15

**Authors:** Masashi Yuki, Reina Aoyama, Masahiro Nakagawa, Takashi Hirano, Eiji Naitoh, Daiki Kainuma

**Affiliations:** Yuki Animal Hospital, 2-99, Kiba-cho Minato-ku, Nagoya, Aichi 4550021, Japan; vv.reina.07@gmail.com (R.A.); nihonto_nakagawamasahiro@yahoo.co.jp (M.N.); hiranoon007@docomo.ne.jp (T.H.); nedved3311@gmail.com (E.N.); cancerdaiki@yahoo.co.jp (D.K.)

**Keywords:** acute phase protein, cats, inflammation, primary care hospital, prognosis, serum amyloid A, survival

## Abstract

Although measurement of serum amyloid A (SAA) concentration in client-owned cats has already been shown to be clinically useful, limited data are available on common diseases at primary care hospitals. In this study, we measured the SAA concentration in cats with various diseases and investigated their clinical significance using a primary care hospital as a population. We measured the SAA concentrations in healthy cats (n = 98) and those with various clinical signs (n = 444). The SAA concentrations in healthy cats did not differ significantly by age, breed, sex, and presence/absence of neutering/spaying. The SAA concentrations were significantly higher in the diseased cat group than in the healthy cat group (*p* < 0.001). We observed significant increases in SAA concentrations in cats with confirmed diagnosis of inflammatory disease such as upper respiratory tract infections (*p* < 0.001), pneumonia (*p* < 0.001), pyometra (*p* = 0.001), and feline infectious peritonitis (*p* < 0.001), compared with those observed in healthy cats. Conversely, no increase was observed in cardiomyopathy, hyperthyroidism, and diabetes mellitus without systemic inflammation. In univariate analysis, survival at 30 days (*p* = 0.03) differed significantly between the low and high SAA concentration groups, but not at 180 days. In multivariate analysis, survival at 30 days did not significantly affect SAA concentration. Measurement of SAA concentration is a useful biomarker for detecting the presence or absence of inflammation in diseased cats. However, it may not be useful as a biomarker for determining the prognosis of the disease.

## 1. Introduction

Acute phase proteins (APPs) are known to include C-reactive protein (CRP), serum amyloid A (SAA), α-1 acid glycoprotein, haptoglobin, and fibrinogen [1]. In humans, SAA is considered to be diagnostically superior to CRP in pathological conditions such as viral diseases, rejection reactions after organ transplantation, and infection-related complications after steroid use [2]. In dogs, SAA is also reported to diagnose a wider range of conditions than that diagnosed by CRP [3].

In cats, SAA concentrations, compared with CRP concentrations, increase markedly in the early stages of diseases, and SAA, with its greater change rate, has been considered useful, and is clinically applied as a biomarker for determining the diagnosis and treatment course of diseases [4,5]. SAA concentrations are known to increase in cats with infection-related diseases, tissue injury, trauma, surgery, tumor, and immune-mediated diseases [1,4,5]. To date, however, the bulk of the SAA concentration data has been obtained from laboratory animals and secondary care hospitals, with limited SAA data in clinical studies obtained from primary care hospitals.

In this study, we hypothesized that data from laboratory animals and secondary care hospitals may be different from that of primary care hospitals, and to demonstrate this hypothesis, we measured SAA concentrations for various client-owned cat diseases and investigated.

## 2. Material and Methods

### 2.1. Animal Ethics

In Japan, there is no ethics committee available for private-practice animal hospitals. Nevertheless, this research was conducted according to the ethical codes of the Japan Veterinary Medical Association. The samples obtained in this study were used after obtaining written consent from each cat owner.

### 2.2. Patient Population

In this prospective observational study at Yuki Animal Hospital, we recruited 98 healthy client-owned cats and 444 client-owned cats with various medical diseases between February 2015 and December 2018. We performed a physical examination, complete blood count (CBC), serum biochemistry profile, feline immunodeficiency virus (FIV) antibody test, and feline leukemia virus (FeLV) antigen test (SNAP FIV/FeLV Combo; IDEXX Laboratories, Maine, USA) and ensured that cats falling under the healthy group had no abnormal results of these examinations. The cats under the diseased group were diagnosed suffering with various diseases through abnormalities detected in these examinations and other relevant examinations conducted.

### 2.3. Blood Collection and Quantification

We collected blood samples for CBC, serum, and plasma analysis from all cats via the venipuncture of the cephalic, saphenous, or jugular vein and placed them in tubes with or without an anticoagulant. Plasma/serum was separated from the blood samples within 30 min of collection. These samples were analyzed on the day of collection.

### 2.4. Assays

We measured CBC using an automated hematology analyzer (Celltac alpha; Nihon Koden, Tokyo, Japan). We obtained a serum biochemical profile using a dry chemistry analyzer (FUJI DRI-CHEM 7000V; FUJIFILM Corporation, Tokyo, Japan). We obtained SAA concentrations using an automated analyzer (turbidimetric immunoassay) (SpeLIA; Precision System Science Co., Ltd., Chiba, Japan). The intra-assay coefficient of variation values was < 15%. Our measured values correlated very well with previously reported values determined using the turbidimetric immunoassay (*y* = 1.0008 *x* + 4.1576, *r* = 0.9641, n = 71 for SAA) [1,5].

### 2.5. Data Analysis and Statistics

Using the Mann–Whitney *U*-test, we compared the SAA concentrations between healthy cats and those in each disease group, and between sexes and between breeds. For the relationships among age, sex and neutered versus non-neutered, and FIV and FeLV infections, we used the Kruskal–Wallis one-way analysis of variance. We generated survival curves using the Kaplan–Meier product-limit method and compared them using the log-rank test. We performed multivariate (logistic regression analysis) analyses on factors likely to affect mortality. We considered values of *p* < 0.05 significant. We performed statistical analyses using Easy R software [6].

## 3. Results

### 3.1. SAA Concentration in Healthy Cats

The healthy cat group comprised 38 females (12 sexually intact, 26 spayed) and 60 males (29 sexually intact, 31 castrated). Breeds in this group comprised mixed breed (n = 89), American Shorthair (n = 3), Scottish Fold (n = 2), Chartreux (n = 1), Norwegian Forest Cat (n = 1), Somali (n = 1), and Ragdoll (n = 1). The median (known) age of healthy cats was 4.95 years (IQR, 0.6–11.2 years); 18 healthy cats were of unknown age. The median body weight was 3.9 kg (IQR, 3.3–4.7 kg). Healthy cats had a median SAA concentration of 0 μg/mL (IQR, 0–0 μg/mL) (Table 1). The median SAA concentration in the group aged under 1 year (n = 28; median, 0 μg/mL; IQR, 0–0 μg/mL), the group aged between 1 and 4 years (n = 12; 0 μg/mL; IQR, 0–0 μg/mL), the group aged between 5 and 9 years (n = 16; 0 μg/mL; IQR, 0–0.41 μg/mL), and the group aged 10 years and over (n = 24; 0 μg/mL; IQR, 0–0 μg/mL), was in each case 0 μg/mL, and the groups did not differ significantly (*p* = 0.94). In addition, SAA concentrations did not differ significantly between sexes (females versus males, *p* = 0.46); sex and neutered versus non-neutered (*p* = 0.42); and breeds (mongrels versus purebreds, *p* = 0.92). From these healthy cats, we selected an age-matched healthy cat group (n = 45; median 0 μg/mL; IQR, 0–0.04 μg/mL) for the diseased cat group.

### 3.2. The SAA Concentration in Diseased Cats

**Figure 1:**
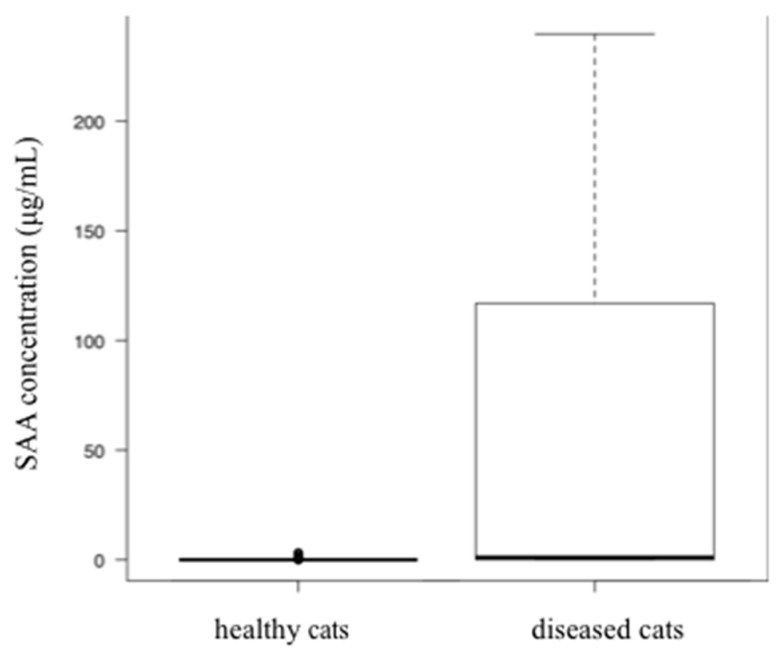
A comparison of SAA concentration between age-matched healthy cats and cats with diseases. The SAA concentrations were significantly higher in the diseased cat group than in the healthy cat group (*p* < 0.001).

The diseased cat group comprised 213 females (45 sexually intact, 168 spayed) and 231 males (71 sexually intact, 160 castrated). Breeds in the diseased group comprised mixed breed (n = 389), American Shorthair (n = 12), Scottish Fold (n = 11), Persian (n = 7), Munchkin (n = 6), Russian Blue (n = 5), American curl (n = 3), Ragdoll (n = 3), Maine Coon (n = 2), Norwegian Forest Cat (n = 2), Bengal (n = 1), Himalayan (n = 1), Siamese (n = 1), and Somali (n = 1). The median (known) age of diseased cats was 10.7 years (IQR, 5.8–14.2 years); 95 of this group were of unknown age. The median body weight was 3.7 kg (IQR, 2.9–4.6 kg). SAA concentrations of the group, including all cats of the diseased cat group, were significantly higher than those in the age-matched healthy cat group (*p* < 0.001) (Figure 1).

Diagnoses in diseased cats included upper respiratory tract infections (n = 37), pneumonia (n = 14), gingivostomatitis (n = 37), gastroenteritis (n = 59), pancreatitis (n = 20), hepatitis/cholangitis (n = 8), chronic kidney disease (n = 83), lower urinary tract diseases (n = 51), cardiomyopathy (n = 9), hyperthyroidism (n = 13), diabetes mellitus (n = 5), pyometra (n = 7), ketoacidosis (n = 8), feline infectious peritonitis (n = 5), traumatic diseases (n = 35), solid tumor (n = 19; malignant mammary gland tumor, pulmonary adenocarcinoma, bile duct tumor, squamous carcinoma, hepatocellular tumor, fibrosarcoma, and hemangiosarcoma), and round cell tumor (n = 30; lymphoma, leukemia, multiple myeloma, and mast cell tumor). The data for each disease group are shown in Table 1.

**Figure 2:**
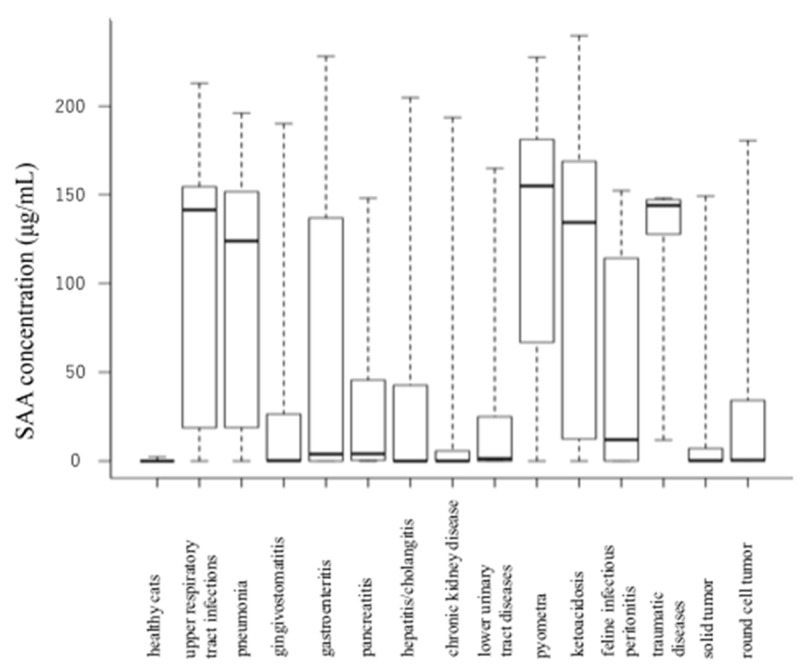
A comparison of SAA concentration between age-matched healthy cats and cats with various diseases (significant difference).

**Figure 3:**
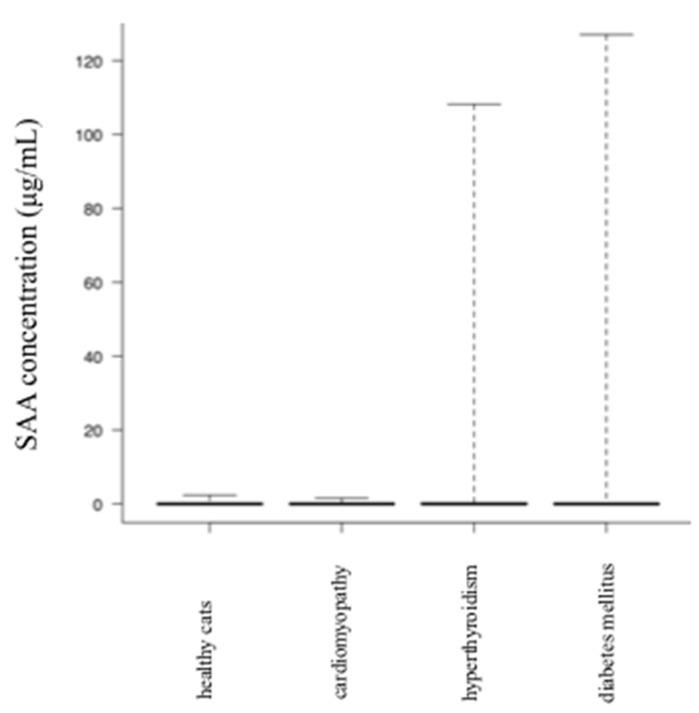
A comparison of SAA concentration between age-matched clinically healthy cats and cats with various diseases (no significant difference).

**Figure 4:**
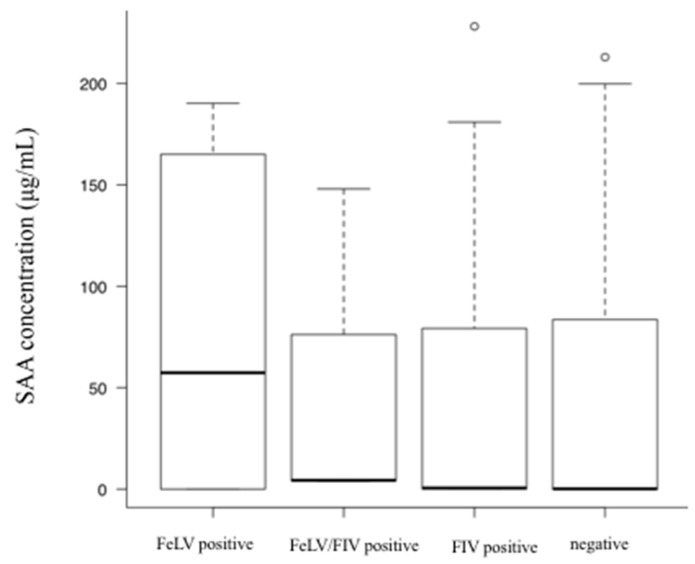
A comparison of SAA concentrations between feline leukemia virus (FeLV) / feline immunodeficiency virus (FIV) negative cats and those with FIV and/or FeLV infection. There was no significant difference (*p* = 0.32) in SAA concentration among each group of FeLV positive, FIV positive, FeLV/FIV positive, and FeLV/FIV negative cats.

The SAA concentrations of cats with upper respiratory tract infections, pneumonia, gingivostomatitis, gastroenteritis, pancreatitis, hepatitis/cholangitis, chronic kidney disease, lower urinary tract diseases, pyometra, ketoacidosis, feline infectious peritonitis, traumatic diseases, solid tumor, and round cell tumor were significantly higher than those in age-matched healthy cats. Results of each disease group vs. healthy cats are shown in Table 1 and Figure 2. The SAA concentrations of cats with cardiomyopathy, hyperthyroidism, and diabetes mellitus did not differ significantly from those in healthy cats (Table 1) (Figure 3). In addition, there was no significant difference (*p* = 0.32) in SAA concentration among each group of FeLV positive (n = 7), FIV positive (n = 40), FeLV/FIV positive (n = 3), and FeLV/FIV negative cats (n = 208) (Figure 4).

### 3.3. SAA Concentration as a Prognostic Factor

We confirmed survival and non-survival during the study period by medical records or interviews with owners. We classified cats into two groups: the low SAA group (n = 222) and the high SAA group (n = 222) with the median SAA concentration as the cutoff value (1.2 μg/mL).

**Figure 5:**
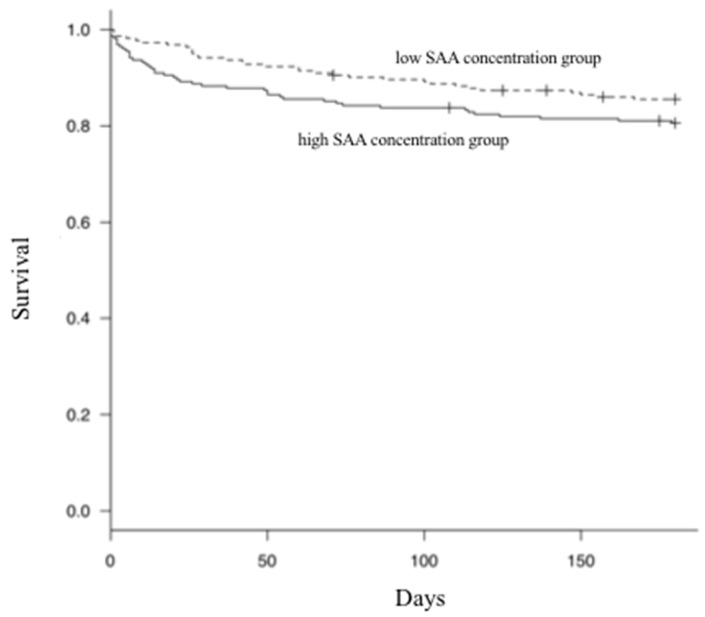
Kaplan–Meier curves of SAA concentration at diagnosis. In the univariate analysis, there was a significant difference in survival at 30 days between the low and high SAA concentration groups (*p* = 0.03), but not at 180 days (*p* = 0.12).

In univariate analysis, survival at 30 days (*p* = 0.03) in the low SAA concentration group differed significantly from that in the high SAA concentration group, but at 180 days (*p* = 0.14), this difference diminished (Figure 5). Based on past studies [5,7,8], we performed multivariate analysis, including 12 factors (age, serum concentration of SAA, albumin, blood urea, nitrogen, creatinine, glucose, alanine transaminase, hematocrit, white blood cell count, neutrophil count, and FeLV/FIV infections) that are likely to be related to prognosis prediction. We found no significant difference in survival at 30 days (*p* = 0.76) irrespective of the SAA concentration.

### 3.4. Relationship between Increase in SAA Concentration after Trauma Disease and Elapsed Time

**Figure 6:**
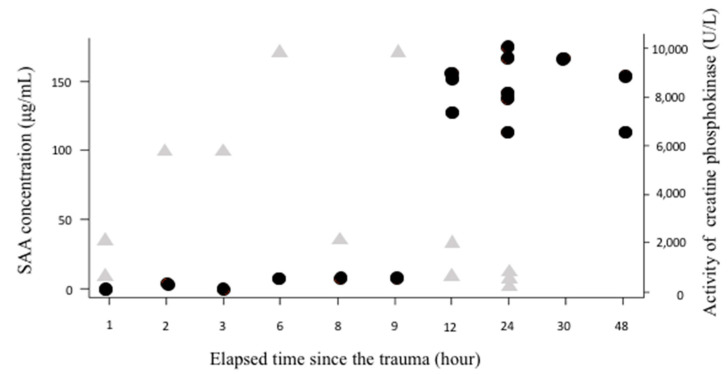
The time taken for SAA concentration to increase in the trauma group. The activity of creatinine phosphokinase (CPK) (triangle marks) increased immediately one and two hours after injury, but the SAA concentration (circle marks) increased 12 h after injury.

We investigated the relationship between the increase in SAA concentration and activity of creatinine phosphokinase (CPK) and elapsed time for trauma cases (n = 25, traffic accident, fall accident and skin injury) where the injury time was clear. The activity of CPK increased immediately one and two hours after injury, but the SAA concentration increased only at 12 h after injury (Figure 6).

## 4. Discussion

In cats, SAA concentration is currently applied clinically as the most sensitive and useful APP because it increases early in the course of inflammation, and at a higher change rate compared with that of other APPs such as CRP and AGP [4]. It is known to be increased in immune-mediated diseases, in tumors with inflammation, in infections, specifically those caused by Hemoplasma spp. [9], as well as in urinary tract disorder, post-surgery [4], pancreatitis [10], injury, infection-related disease, feline lower urinary tract disease, tumor, hyperthyroidism, diabetes mellitus [5,11,12], renal disease [5,7,13,14], mammary tumors [15], lymphoma, mesothelioma [16], feline infectious peritonitis [5,7,17,18], and sepsis [19], and is reported to be useful as a diagnostic aid. These reports include endocrine diseases not generally associated with inflammation, such as hyperthyroidism or diabetes mellitus. The results of our study showed that in hyperthyroidism and diabetes mellitus, there was no significant increase in SAA concentration compared with that of healthy cats. The difference between these results was that most of the previous reports investigated data from the secondary care hospital, which may have included complex cases involving inflammation, where the concentration of SAA would have increased. Moreover, in this study, it was possible that the results differed because we separated diabetes and ketoacidosis and excluded cases with concurrent disease where possible.

Elevated SAA concentrations were observed in chronic kidney disease, consistent with previous reports [14]. Many patients with azotemia had significant increases in pro-inflammatory levels of cytokines such as IL-6 and IL-12 in circulation, when compared with those of healthy controls [20]. Similarly, chronic kidney disease in cats is predicted to increase SAA concentration due to the increase of these cytokines. On the other hand, although circulating TNF-α and IL-6 levels are known to increase in human cardiomyopathy [21], this study did not detect an increase in SAA concentration in cardiomyopathy. This result is difficult to interpret, and may be due to the small number of cases; future studies involving the measurement of circulating inflammatory cytokine levels in cats with cardiomyopathy are indicated.

The diseased cats in this study included cases infected with FeLV and FIV. However, while these infected cats were not associated with increased SAA concentrations, previous studies have reported elevated SAA concentration in cats with clinical signs of FIV infection. On the other hand, almost no elevation has been demonstrated in cats without clinical signs [8]. In this study, no FIV-infected cats showed any clinical signs, which may have resulted in FIV infection not being associated with elevated SAA concentrations. There are no similar studies in FeLV-infected cats, but our results may be attributed to the same reason as that for FIV-infected cats. Further studies are needed to determine the relationship between these infections and SAA concentration.

In a report from a secondary care hospital, SAA was reported to be useful as a prognostic marker because the survival time of the high SAA concentration group was significantly shorter than that of the normal concentration group [5]. However, in our multivariate analysis, SAA concentration was not a predictive factor for survival at day 30. The population of this study was from a primary care hospital, and there were many cases where the concentration of SAA was high in diseases of mild severity, such as upper respiratory tract infections. Data for populations at secondary care hospitals are unlikely to include diseases of mild severity, such as upper respiratory tract infections. Although a clear difference exists between these two populations, the measurement of SAA concentrations, at least in the primary care hospitals, may not be useful as a prognostic marker for, for instance, survival time. In the future, studies ascertaining the efficacy of SAA measurement as a prognostic marker for each disease will be needed.

The activity of CPK, known to increase after muscle damage due to trauma, increased soon after injury [22], whereas the SAA concentration increased with a delay of about 10 h after injury. Previous reports, consistent with the results of this study, demonstrated that SAA concentrations began to increase gradually at 8 h after tissue damage, peaking at 2 days [4]. SAA, induced by inflammatory cytokines such as IL-1, IL-6, and TNF, which increase when acute inflammation occurs, is synthesized in the liver, resulting in a time lag before its increase in circulation [1]. It is important to understand the characteristics of SAA that are different from deviant enzymes that increase immediately after such tissue destruction.

It has already been reported that SAA concentrations do not differ between male and female cats [4], consistent with the results of this study; in addition, we have confirmed here that the SAA concentrations are not related to age and presence or absence of neutering. Regarding breeds, there is no significant difference in SAA concentration between mongrels and purebreds, but in the absence of a survey among purebreds, this topic needs to be examined in the future.

This study has some limitations. First, while the SAA concentration for each disease should be measured at its peak, in practice this is difficult; therefore, measurement errors are always included. Second, the number of cases in the evaluation for each disease is frequently small, and cases where the sample power is not statistically sufficient may be included. Third, concurrent disease may not have been completely ruled out, leading to an incorrect result. Finally, this study did not exclude hemolysis, bilirubinemia, and lipemia specimens. While ELISA has been confirmed as not being affected by these effects [1], turbidimetric immunoassay has not been likewise confirmed, which may have influenced the results of this study.

## 5. Conclusions

The measurement of SAA concentrations in a primary care hospital revealed an increase in tandem with disease with inflammation and the characteristics of APPs. Moreover, it was not useful as a marker for predicting prognosis. In the future, the efficacy of SAA concentration measurement in diagnosing each disease should be examined.

## Figures and Tables

**Table 1:** The serum amyloid A (SAA) concentration in age-matched healthy cats and cats with various diseases.

Diagnosis	No.	SAA Concentration		Healthy vs Disease Cat
		Median (μg/mL)	IQR (μg/mL)	*p* Value
Age-matched-healthy cats	n = 45	0	0–0.004	
Upper respiratory tract infections	n = 41	141.4	17.9–155.4	<0.001
Pneumonia	n = 14	134.3	12.5–168.7	<0.001
Gingivostomatitis	n = 37	1.3	0–52.8	<0.001
Gastroenteritis	n = 59	0.3	0–29.0	<0.001
Pancreatitis	n = 20	3.9	0–138.1	0.003
Hepatitis/cholangitis	n = 8	12.0	0–123.5	0.005
Chronic kidney disease	n = 83	0.03	0–5.9	0.002
Lower urinary tract diseases	n = 51	0	0–53.8	0.02
Pyometra	n = 7	154.8	0.1–182.4	0.001
Ketoacidosis	n = 8	4.1	0.3–60.5	<0.001
Feline infectious peritonitis	n = 5	143.9	69.8–147.5	<0.001
Traumatic diseases	n = 35	123.9	18.8–152.3	<0.001
Solid tumor	n = 19	0.3	0–10.7	0.005
Round cell tumor	n = 30	0.5	0–42.7	0.005
Cardiomyopathy	n = 9	0	0–2.9	0.88
Hyperthyroidism	n = 13	0	0–20.4	0.54
Diabetes mellitus	n = 5	0	0–0.8	0.62
